# Comparison of Novel and Established Nitrification Inhibitors Relevant to Agriculture on Soil Ammonia- and Nitrite-Oxidizing Isolates

**DOI:** 10.3389/fmicb.2020.581283

**Published:** 2020-11-04

**Authors:** Evangelia S. Papadopoulou, Eleftheria Bachtsevani, Eleni Lampronikou, Eleni Adamou, Afroditi Katsaouni, Sotirios Vasileiadis, Cécile Thion, Urania Menkissoglu-Spiroudi, Graeme W. Nicol, Dimitrios G. Karpouzas

**Affiliations:** ^1^Laboratory of Plant and Environmental Biotechnology, Department of Biochemistry and Biotechnology, University of Thessaly, Larissa, Greece; ^2^Laboratoire Ampère, École Centrale de Lyon, University of Lyon, Ecully, France; ^3^Pesticide Science Laboratory, School of Agriculture, Forestry and Environment, Faculty of Agriculture, Aristotle University of Thessaloniki, Thessaloniki, Greece

**Keywords:** nitrification inhibitors, ammonia-oxidizing bacteria, ammonia-oxidizing archaea, nitrite-oxidizing bacteria, ethoxyquin, quinone imine

## Abstract

Nitrification inhibitors (NIs) applied to soil reduce nitrogen fertilizer losses from agr*o*-ecosystems. NIs that are currently registered for use in agriculture appear to selectively inhibit ammonia-oxidizing bacteria (AOB), while their impact on other nitrifiers is limited or unknown. Ethoxyquin (EQ), a fruit preservative shown to inhibit ammonia-oxidizers (AO) in soil, is rapidly transformed to 2,6-dihydro-2,2,4-trimethyl-6-quinone imine (QI), and 2,4-dimethyl-6-ethoxy-quinoline (EQNL). We compared the inhibitory potential of EQ and its derivatives with that of dicyandiamide (DCD), nitrapyrin (NP), and 3,4-dimethylpyrazole-phosphate (DMPP), NIs that have been used in agricultural settings. The effect of each compound on the growth of AOB (*Nitrosomonas europaea, Nitrosospira multiformis*), ammonia-oxidizing archaea (AOA; “*Candidatus* Nitrosocosmicus franklandus,” “*Candidatus* Nitrosotalea sinensis”), and a nitrite-oxidizing bacterium (NOB; *Nitrobacter* sp. NHB1), all being soil isolates, were determined in liquid culture over a range of concentrations by measuring nitrite production or consumption and qPCR of *amoA* and *nxrB* genes, respectively. The degradation of NIs in the liquid cultures was also determined. In all cultures, EQ was transformed to the short-lived QI (major derivative) and the persistent EQNL (minor derivative). They all showed significantly higher inhibition activity of AOA compared to AOB and NOB isolates. QI was the most potent AOA inhibitor (EC_50_ = 0.3–0.7 μM) compared to EQ (EC_50_ = 1–1.4 μM) and EQNL (EC_50_ = 26.6–129.5 μM). The formation and concentration of QI in EQ-amended cultures correlated with the inhibition patterns for all isolates suggesting that it was primarily responsible for inhibition after application of EQ. DCD and DMPP showed greater inhibition of AOB compared to AOA or NOB, with DMPP being more potent (EC_50_ = 221.9–248.7 μM *vs* EC_50_ = 0.6–2.1 μM). NP was the only NI to which both AOA and AOB were equally sensitive with EC_50s_ of 0.8–2.1 and 1.0–6.7 μM, respectively. Overall, EQ, QI, and NP were the most potent NIs against AOA, NP, and DMPP were the most effective against AOB, while NP, EQ and its derivatives showed the highest activity against the NOB isolate. Our findings benchmark the activity range of known and novel NIs with practical implications for their use in agriculture and the development of NIs with broad or complementary activity against all AO.

## Introduction

Modern agricultural systems depend heavily on large inputs of synthetic N fertilizers to maintain crop productivity and meet the increasing global food demand (Fowler et al., 2018). However, *ca*. 70% of the annual global input of 100 Tg N fertilizer is lost from agricultural ecosystems due to nitrification and subsequent denitrification processes leading to groundwater and atmospheric pollution (Raun and Johnson, 1999). To minimize N losses and improve N use efficiency in soil, nitrification inhibitors (NIs), compounds known to reduce the activity of nitrifying prokaryotes, are routinely incorporated into N-stabilized fertilizers (Abbasi and Adams, 1998; Moir et al., 2007).

Hundreds of compounds have been identified that inhibit nitrifying prokaryotes (Bédard and Knowles, 1989; McCarty, 1999) including plant-derived molecules (Coskun et al., 2017), aliphatic and aromatic n-alkynes (Taylor et al., 2015; Wright et al., 2020), statins (Zhao et al., 2020), and PTIO (2-phenyl-4,4,5,5-tetramethylimidazoline-1-oxyl 3-oxide; Martens-Habbena et al., 2015). Many of these are used as selective inhibitors of ammonia-oxidizing bacteria (AOB; e.g., octyne) or archaea (AOA; e.g., PTIO) in laboratory cultures, soil microcosms or slurries, but are not suitable for use in an agricultural setting due to rapid degradation in soil or application in a gaseous state. Only three compounds have gained importance for practical use as NIs in agriculture: 2-chloro-6-(trichloromethyl) pyridine (nitrapyrin; NP; Goring, 1962), dicyandiamide (DCD; Solansky, 1982), and 3,4-dimethylpyrazole phosphate (DMPP; Zerulla et al., 2001). All three are presumed to act as Cu chelators interfering with ammonia monooxygenase (AMO), a key enzyme in the first and rate-limiting step of nitrification (Ruser and Schulz, 2015). In addition, NP was also proposed to serve as a weak mechanism-based or “suicide” inhibitor (Vannelli and Hooper, 1992). However, the precise mode of action of these NIs has yet to be fully elucidated.

When NIs were first introduced in agriculture, soil nitrification was considered a two-step process carried out by AOB and nitrite-oxidizing bacteria (NOB). AOB oxidize ammonia to hydroxylamine (NH_2_OH) using AMO, which is further oxidized to nitric oxide (NO) and finally nitrite (NO_2_^–^). NOB subsequently transform NO_2_^–^ to nitrate (NO_3_^–^) using nitrite oxidoreductase (NXR; Coskun et al., 2017; Beeckman et al., 2018). However, over the last 15 years, other groups were demonstrated to contribute to soil nitrification including AOA (Leininger et al., 2006; Zhang et al., 2012), and recently “comammox” *Nitrospira*, (Wang et al., 2019; Li et al., 2019a) that perform complete oxidation of ammonia to nitrate within an individual cell (Daims et al., 2015; van Kessel et al., 2015). Isolation of soil AOA strains confirmed their role in soil ammonia oxidation (Tourna et al., 2011; Lehtovirta-Morley et al., 2014), while all *Nitrospira* strains isolated from soil are non-comammox strains.

Despite these breakthroughs in our understanding of the microbiology and biochemistry of nitrification, current knowledge regarding the spectrum of activity and the inhibition thresholds of NIs used in agriculture on soil ammonia-oxidizers (AO) is limited. The use of inhibition assays with pure cultures of a diverse range of soil-derived strains is a necessary benchmarking step to define the exact spectrum of activity of NIs destined for use in agriculture. Most culture inhibition assays have focused on AOB (e.g., Bélser and Schmidt, 1981; Vannelli and Hooper, 1992) or tested NIs not broadly applied in agricultural settings on soil AOA strains (i.e., allylthiourea, n-aliphatic alkynes, and simvastatin; Wright et al., 2020; Zhao et al., 2020). Others have explored the activity of NIs of agricultural relevance on AOA (Jung et al., 2011; Kim et al., 2012), but only three provided a systematic assessment and inhibition thresholds for AOA soil strains like “*Candidatus* Nitrosocosmicus agrestis” (Liu et al., 2019), “*Candidatus* Nitosotalea devanaterra” (Lehtovirta-Morley et al., 2013), and *Nitrososphaera viennensis* (Shen et al., 2013). In addition, most NIs are known to act on the ammonia oxidation step of nitrification (Bédard and Knowles, 1989), hence their activity on NOB remains unknown. The variation in sensitivity of AOA and AOB to different types of NIs, combined with their contribution to nitrification in distinct ecological niches (Prosser and Nicol, 2012; Kits et al., 2017), implies a potential suboptimal efficiency of the NIs currently used in agriculture, and stresses the need for the discovery of novel NIs with a broader range of activity against all microorganisms contributing to nitrification.

In previous soil microcosm studies we showed that ethoxyquin (EQ; 1,2-dihydro-6-ethoxy-2,2,4-trimethylquinoline), an antioxidant used as preservative in fruit-packaging plants, and its derivative 2,6-dihydro-2,2,4-trimethyl-6-quinone imine (QI), strongly inhibited the activity of AOB and AOA (Papadopoulou et al., 2016). EQ in soil is rapidly transformed to QI and 2,4-dimethyl-6-ethoxyquinoline (EQNL; Karas et al., 2015). The potential capacity of EQ to be rapidly transformed in soil to potent NIs is of particular interest, considering that the spectrum and the duration of inhibition are desirable attributes of NIs used in agricultural practice.

We aimed to determine the inhibitory potency of EQ and its derivatives on representative isolates of diverse and globally distributed lineages of soil AOB and AOA in liquid culture, in comparison to NIs widely used in agricultural settings (NP, DCD, and DMPP). We expanded our liquid inhibition assays to NOB to gain insights on the impact of NIs on a microbial group functionally associated with ammonia oxidation, and directly linked to nitrogen loss from disturbed agricultural ecosystems in the form of nitrate production. Specifically, we used (i) AOB strains *Nitrosomonas europaea* and *Nitrosospira multiformis*, belonging to AOB clusters 7 and 3, respectively, (Purkhold et al., 2000), with cluster 3 often being the dominant AOB lineage in soil ecosystems (Kowalchuk and Stephen, 2001); (ii) AOA strains “*Candidatus* Nitrosocosmicus franklandus” (Lehtovirta-Morley et al., 2016) and “*Candidatus* Nitrosotalea sinensis” (Lehtovirta-Morley et al., 2014), occupying contrasting ecological niches and representing widely distributed neutrophilic and acidophilic AOA lineages, respectively, (Herbold et al., 2017), and (iii) one NOB, *Nitrobacter* sp. NHB1 (de Boer et al., 1991) as a representative of one of the two dominant NOB lineages found in soil (Daims et al., 2016), with *Nitrobacter* strains typically having greater nitrite oxidation activity compared to *Nitrospira*, and dominating activity under excess nitrogen supply (e.g., fertilized soils; Xia et al., 2011; Nowka et al., 2015). While previous studies have examined the effective concentration of different NIs on ammonia oxidizer isolates, this study also examined the degradation of NIs during laboratory incubation.

## Materials and Methods

### Microbial Strains, Growth Conditions and Chemicals

All strains were grown aerobically in the dark without shaking. AOB *N. europaea* ATCC25978 and *N. multiformis* ATCC25196 were grown at 28°C in Skinner and Walker’s medium (Skinner and Walker, 1961) containing 1 mM NH_4_^+^ [(NH_4_)_2_SO_4_] and phenol red (0.5 mg L^–1^) as a pH indicator. AOA “*Ca.* N. franklandus” C13 and “*Ca.* N. sinensis” ND2, were incubated at 35°C in a medium supplemented with 1 mM NH_4_^+^ (NH_4_Cl). The former was cultured in HEPES-buffered modified freshwater medium (pH 7.5; Lehtovirta-Morley et al., 2014), while the latter was grown in freshwater medium (pH 5.2; Lehtovirta-Morley et al., 2011). *Nitrobacter* sp. strain NHB1 was grown at 28°C in freshwater medium (pH 5.2; Lehtovirta-Morley et al., 2011) supplemented with 0.5 mM NO_2_^–^ (NaNO_2_).

Analytical standards of DCD (99% purity), NP (≥98%), and EQ (95%) were purchased from Sigma-Aldrich (Germany), while DMPP (99.1%) analytical standard was provided by BASF Hellas. The oxidation derivatives of EQ, QI, and EQNL were synthesized as described by Thorisson et al. (1992). The chemical structures of all studied compounds are shown in Supplementary Figure 1.

### Liquid Culture Assays

The activity of all NIs was determined in liquid batch cultures over a range of concentrations to establish relevant inhibition thresholds per strain and compound. Preliminary assays with a broad range of concentrations for each NI and isolate (NO_2_^–^ production) dictated the range of NI concentrations that will allow calculation of inhibition thresholds. Cultures were established in triplicate for each strain × NI × concentration combination in 100-mL Duran bottles containing 50 mL of growth medium and inoculated with a 1 or 2% (v/v) transfer of exponentially growing cultures of AOB or AOA/NOB, respectively. EQ, QI, EQNL, and NP were added to the cultures as filter sterilized dimethyl sulfoxide (DMSO) solutions due to their low water solubility (≤60 mg L^–1^ at 20°C). The final concentration of DMSO in all cultures was 0.1% (v/v), which did not exert a significant inhibitory effect to any of the isolates tested (data not shown), in line with previous studies with the same isolates (Wright et al., 2020; Zhao et al., 2020). DCD and DMPP were dissolved in sterile dH_2_O before addition of 25 μl (0.5% v/v). All NIs were added to batch cultures at the beginning of the exponential growth phase. For all assays, triplicate cultures with the same inoculum not amended with NIs were included. Upon inoculation all liquid batch cultures were sampled at regular time intervals to determine the effect of NIs on the activity and growth of nitrifying microorganisms by measuring changes in nitrite concentrations and the abundance of *amoA* (AO) or *nxrB* (NOB) genes, respectively.

### Nitrite Measurements and Gene Abundance Quantification

Nitrite concentrations were determined colorimetrically at 540 nm in a 96-well plate format assay by diazotizing and coupling with Griess reagent (Shinn, 1941). *amoA* and *nxrB* gene abundance was determined in a Biorad CFX Real–Time PCR system. DNA was extracted from a cell pellet obtained from 2-ml aliquots of the microbial cultures using the tissue DNA extraction kit (Macherey-Nagel, Germany). The *amoA* genes of AOB and AOA was amplified with primers amoA-1F/amoA-2R (Rotthauwe et al., 1997) and Arch-amoAF/Arch-amoAR (Francis et al., 2005), respectively, as described by Rousidou et al. (2013), and the *nxrB* gene of *Nitrobacter* was quantified with primers nxrB-1F and nxrB-1R (Vanparys et al., 2007). All qPCR assays used the following thermal cycling conditions: 95°C for 3 min, followed by 40 cycles of 95°C for 30 s, 57°C for 20 s, 72°C for 30 s, with a final dissociation curve analysis. The abundance of *amoA* and *nxrB* genes were determined via external standard curves as described by Rousidou et al. (2013). qPCR amplification efficiencies ranged from 80.3% to 109.4%, with *r*^2^ values ≥ 0.98.

### Nitrification Inhibitors Extraction

Ethoxyquin, QI, EQNL, and NP residues were extracted from liquid media by mixing 0.3 mL liquid culture with 0.7 mL of acetonitrile. Residues of DCD and DMPP were extracted by mixing 0.1 mL liquid culture with 0.9 mL of ddH_2_O water and methanol, respectively. The derived mixtures were vortexed for 30 s and stored at −20°C until analysis. Recovery tests at three concentration levels (in the range of the tested concentrations) showed recoveries of >80% for all compounds studied.

### Chromatographic Analyses

High performance liquid chromatography (HPLC) analyses were performed in a Shimadzu LC-20ADHPLC system equipped with an UV/VIS PDA detector. A Shimadzu GVP-ODs (4.6 mm by 150 mm, 5 μm) pre-column, connected to a RP Shimadzu VP-ODs (4.6 mm × 150 mm, 5 μm) column, was used for NI separation. The injection volume was 20 μl. The flow rate of the mobile phase was set at 0.8 mL min^–1^ for DCD and at 1 mL min^–1^ for all other NIs. Column temperature was set at 40°C for DCD and DMPP, and at 25°C for all the other NIs. Mixtures of acetonitrile and ammonia [0.25% (vol/vol)] or *ortho*-phosphoric acid [0.1% (vol/vol)] were used at a ratio of 70:30 (vol/vol) for mobile phases in the analyses of EQ, QI, EQNL, and NP, respectively, and detection was achieved at 225, 245, 230, and 269 nm, respectively. Similarly, chromatographic separation of DCD and DMPP was achieved using ddH_2_O (100%) and a mixture of methanol and *ortho*-phosphoric acid [0.1% (vol/vol)] solution 50:50 by volume, respectively. DCD and DMPP residues were detected at 218 nm and 225 nm, respectively.

### Calculation of Inhibition Threshold Levels (EC_50_)

In this study, EC_50_ describes the concentration of the inhibitor that reduces half of the activity (nitrite accumulation or consumption) of AO or NOB. Dose-response modeling was performed using normalized data whereby nitrite concentration values were divided by the mean value of the matching control. Analyses were carried out using the dose response curves (drc) v3.0-1 package (Ritz and Streibig, 2005) of the R software (R Core Team, 2020). A brief description of the tested models can be found in Ritz et al. (2016). An empirical modeling approach was initially used for selecting the best fitting model according to tested goodness of fit indices (see Supplementary Material), followed by the choice of the four-parameter log logistic model as the best compromise among tested models for comparing endpoint values.

### Data Analysis

Nitrite and qPCR data were subjected to one-way ANOVA, followed by Tukey’s *post hoc* test (*P* < 0.05). Variance between the EC_50_ values of the different NIs for one strain and between different strains for a given NI was analyzed by one-way ANOVA, and Duncan *post hoc* test (*P* < 0.05). The four kinetic models proposed by the FOCUS working group on pesticide degradation kinetics (FOCUS, 2006) [single first order kinetic (SFO), biphasic models hockey stick (HS), first order multi-compartment (FOMC), and double first order in parallel (DFOP) models] were used to calculate NI degradation kinetic parameters (DT_50_, k_deg_). Curve fitting was performed with the mkin v0.9.47.1 package (Ranke, 2018) of the R Studio v4.0.2 software (R Core Team, 2020).

## Results

### The Impact and Degradation of EQ, QI, and EQNL in AO and NOB Cultures

#### Effects on the Activity and Growth of AO and NOB Isolates

**FIGURE 1:**
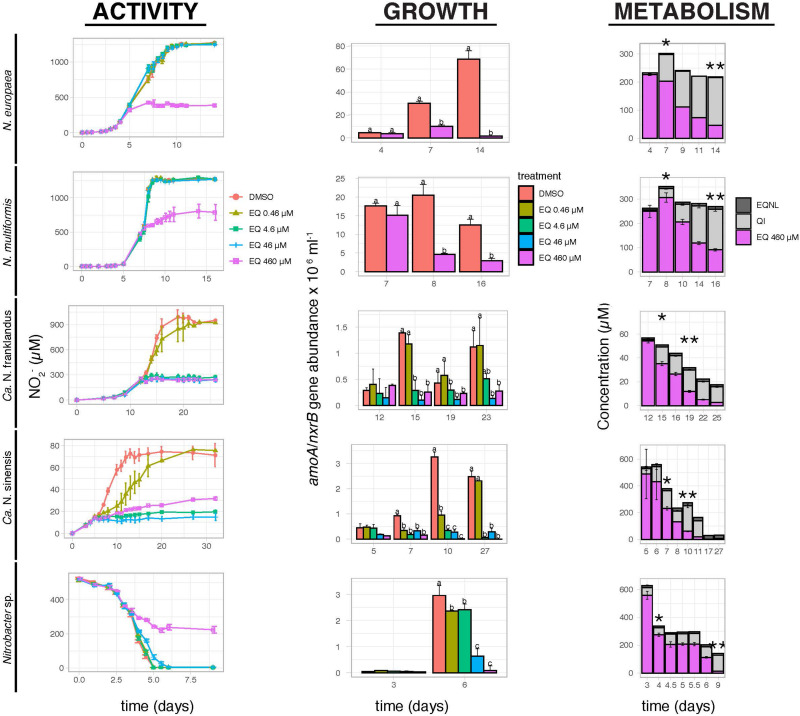
The effect of different concentrations of EQ on the activity and growth of AOB *N. europaea* and *N. multiformis*, AOA “*Ca.* N. franklandus” and “*Ca.* N. sinensis” and NOB *Nitrobacter* sp. NHB1, determined by nitrite production or consumption and the abundance of *amoA* or *nxrB* genes. The degradation and transformation patterns of EQ at the maximum tested concentration (460 μM) are also presented. Error bars represent the standard error of the mean of triplicate cultures. Within each time point, bars designated by different lower-case letters are significantly different at the 5% level. One asterisk indicates the concentrations (μM) of EQ and its oxidative derivatives at the onset of inhibition, while two asterisks indicate the time point when maximum QI concentrations (μM) were observed.

Ethoxyquin fully inhibited the activity of *N. europaea*, *N. multiformis*, and *Nitrobacter* sp. NHB1 only at the highest tested concentration of 460 μM (Figure 1). In contrast, the activity of “*Ca.* N. franklandus” and *“Ca.* N. sinensis” was significantly reduced by EQ at concentrations ≥4.6 μM and ≥0.46 μM (*p* ≤ 0.05), respectively, while complete inhibition of “*Ca*. N. sinensis” was evident at levels ≥4.6 μM. Growth inhibition profiles of AO isolates corresponded with NO_2_^–^ production (Figure 1). In contrast to activity measurements, a significant reduction (*p* ≤ 0.05) in the growth of *Nitrobacter* sp. NHB1 was observed at the end of the incubation period for all EQ concentration levels.

**FIGURE 2:**
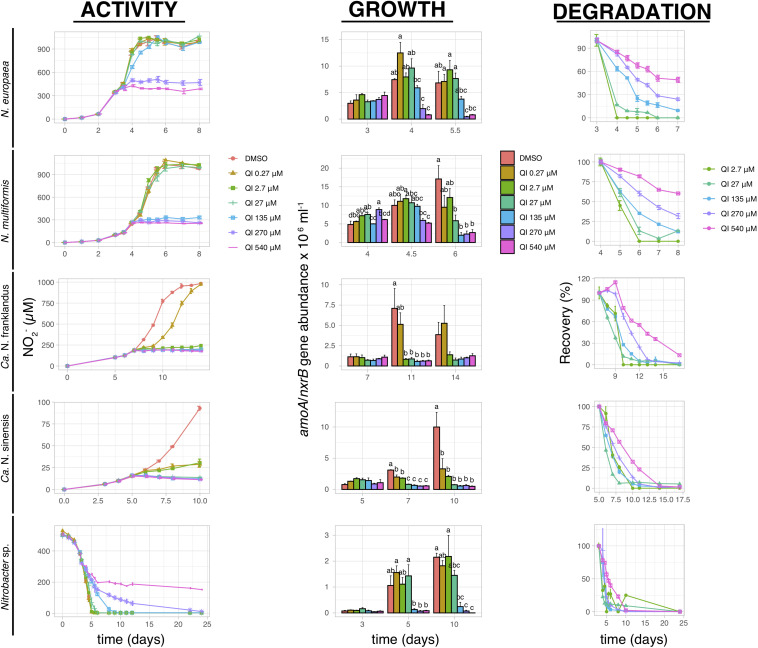
The effect of different concentrations of QI on the activity and growth of AOB *N. europaea* and *N. multiformis*, AOA “*Ca.* N. franklandus” and “*Ca.* N. sinensis” and NOB *Nitrobacter* sp. NHB1, determined by nitrite production, or consumption and the abundance of *amoA* or *nxrB* genes. The degradation pattern of QI applied over a range of concentrations is also presented. Error bars represent the standard error of the mean of triplicate cultures. At each time point, bars designated by different lower-case letters are significantly different at the 5% level.

2,6-dihydro-2,2,4-trimethyl-6-quinone imine fully inhibited ammonia oxidation by *N. europaea* and *N. multiformis* at concentrations ≥270 μM and ≥135 μM, respectively, (Figure 2). The activity of AOA was significantly reduced (*p* ≤ 0.05) at all QI concentrations, with little or no activity at concentrations ≥2.7 μM, and a gradual recovery observed only for “*Ca.* N. franklandus” at the lowest concentration level (0.27 μM). Nitrite consumption by *Nitrobacter* sp. NHB1 was significantly suppressed at concentrations ≥135 μM (*p* ≤ 0.05), though a persistent inhibitory effect was evident only at 540 μM. The inhibition of QI on AO growth concurred with the NO_2_^–^ production patterns, unlike NOB where QI persistently inhibited the growth of *Nitrobacter* sp. NHB1 at concentrations ≥135 μM (Figure 2).

2,4-dimethyl-6-ethoxyquinoline only temporarily inhibited *N. europaea* activity at the highest concentration tested, 500 μM, while at the same concentration level *N. multiformis* activity was fully inhibited (Supplementary Figure 2). Ammonia oxidation by “*Ca.* N. franklandus” and *“Ca.* N. sinensis” was significantly reduced at concentrations ≥125 μM and ≥25 μM (*p* ≤ 0.05), respectively, and complete inhibition occurred at 500 μM and ≥125 μM, respectively. Nitrite oxidation by *Nitrobacter* sp. NHB1 was completely inhibited by EQNL only at the highest tested concentrations of 500 μM (Supplementary Figure 2). While the inhibition of AOB growth was congruent with NO_2_^–^ production, the impact of EQNL on the growth of “*Ca.* N. franklandus” was not fully consistent with the activity measurements, and no significant differences among the different concentrations were observed at the end of the incubation period (day 22), probably due to the decreased number of living cells at EQNL concentrations ≤25 μM. Variations in the growth inhibition pattern of EQNL was observed also for *Nitrobacter* sp. NHB1 which was not significantly (*p* = 0.063) affected by EQNL even at the highest tested concentration (500 μM; Supplementary Figure 2).

#### Degradation Patterns of EQ, QI, and EQNL in Liquid Culture

In the liquid cultures of all tested isolates, EQ was rapidly transformed to QI and EQNL (Figure 1 and Supplementary Figure 3). QI and EQNL constituted 10.4–34.9% and 1.1–4.5%, respectively, of the total amount of EQ recovered at the onset of inhibition in the liquid cultures amended with the highest concentration of EQ (460 μM; Figure 1). The degradation half-life (DT_50_) for the sum of EQ + QI + EQNL in cultures supplemented with 460 μM of EQ ranged from 2.1 days for *Nitrobacter* sp. NHB1 to 60.1 days for *N. multiformis* (Supplementary Table 1).

The degradation of QI, when added directly into liquid culture, was best described by the SFO kinetic model (*x*^2^ ≤ 15, *r*^2^ ≥ 0.75). QI showed limited persistence and a weak dose-dependent degradation pattern with DT_50_ = 0.05–1.52 days at the lowest concentration level (2.7 μM), and 2.23–5.65 days at the highest concentration level (540 μM; Figure 2 and Supplementary Table 1). In contrast, EQNL persisted in the liquid cultures throughout the experiment (extrapolated DT_50_ > 1000 days; Supplementary Figure 2 and Supplementary Table 1).

### The Impact and Degradation of DCD on AO and NOB Cultures

**FIGURE 3:**
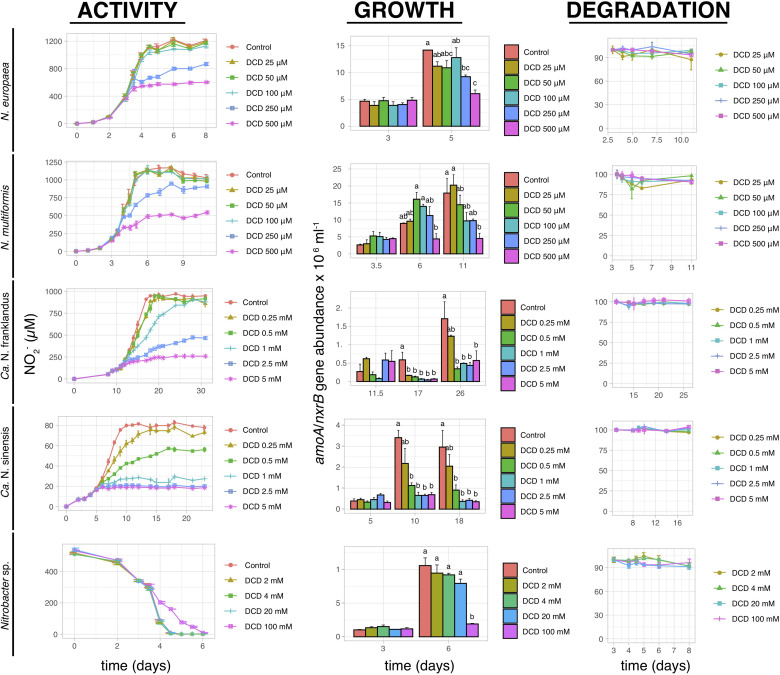
The effect of different concentrations of DCD on the activity and growth of AOB *N. europaea* and *N. multiformis*, AOA “*Ca.* N. franklandus” and “*Ca.* N. sinensis” and NOB *Nitrobacter* sp. NHB1, determined by nitrite production, or consumption and the abundance of *amoA* or *nxrB* genes. The degradation pattern of DCD applied over a range of concentrations is also provided. Error bars represent the standard error of the mean of triplicate cultures. At each time point, bars designated with different lower-case letters are significantly different at the 5% level.

Dicyandiamide significantly inhibited (*p* < 0.05) the activity of both AOB strains at concentrations of 250 μM and 500 μM, with complete inhibition observed only at 500 μM (Figure 3). These concentrations had a reduced or no effect on the two AOA strains, with the activity of “*Ca.* N. franklandus” and *“Ca.* N. sinensis” being significantly inhibited (*p* < 0.05) at concentrations ≥1 mM and ≥0.5 mM, respectively. However, persistent inhibition was evident only at concentrations ≥2.5 mM and ≥1 mM, respectively, (Figure 3). Nitrite oxidation by *Nitrobacter* sp. was significantly inhibited (*p* < 0.05) by DCD only at the highest concentration tested (100 mM). The growth inhibition patterns of AOB and NOB were congruent with the NO_2_^–^ production patterns. This was not the case for AOA where “*Ca.* N. franklandus” growth was significantly reduced at 0.5 mM (*p* ≤ 0.05; Figure 3). DCD did not show a dose-dependent degradation pattern and was rather persistent with DT_50_ values ranging from 45.9 to >1000 days (Figure 3 and Supplementary Table 1).

### The Impact and Degradation of NP on AO and NOB Cultures

**FIGURE 4:**
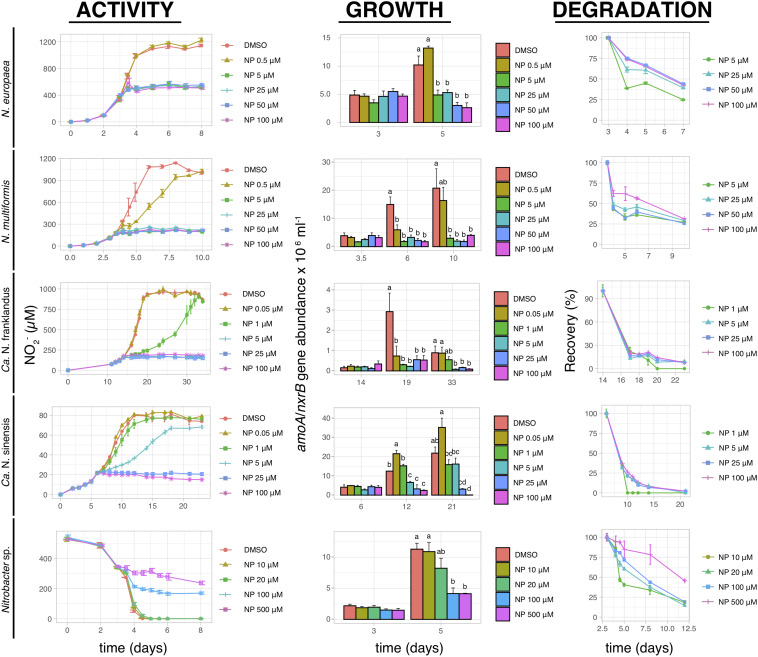
The effect of different concentrations of NP on the activity and growth of AOB *N. europaea* and *N. multiformis*, AOA “*Ca.* N. franklandus” and “*Ca.* N. sinensis” and NOB *Nitrobacter* sp. NHB1, determined by nitrite production, or consumption and the abundance of *amoA* or *nxrB* genes. The degradation pattern of NP applied over a range of concentrations is also presented. Error bars represent the standard error of the mean of triplicate cultures. At each time point, bars designated with different lower-case letters are significantly different at the 5% level.

Nitrapyrin completely inhibited the activity of both *N. europaea* and *N. multiformis* at concentrations ≥5 μM (Figure 4). The activity of “*Ca.* N. franklandus” and *“Ca.* N. sinensis” was significantly reduced at concentrations ≥1 μM and ≥5 μM (*p* ≤ 0.05), with complete inhibition observed at ≥5 μM and ≥25 μM, respectively, (Figure 4). The activity of *Nitrobacter* sp. NHB1 was fully suppressed at concentrations ≥100 μM (Figure 4). The growth inhibition patterns of all tested isolates concurred with the NO_2_^–^ production patterns. NP rapidly degraded in all liquid cultures with DT_50_ values ranging from 0.12 to 12.5 days (Figure 4 and Supplementary Table 1).

### The Impact and Degradation of DMPP on AO and NOB Cultures

**FIGURE 5:**
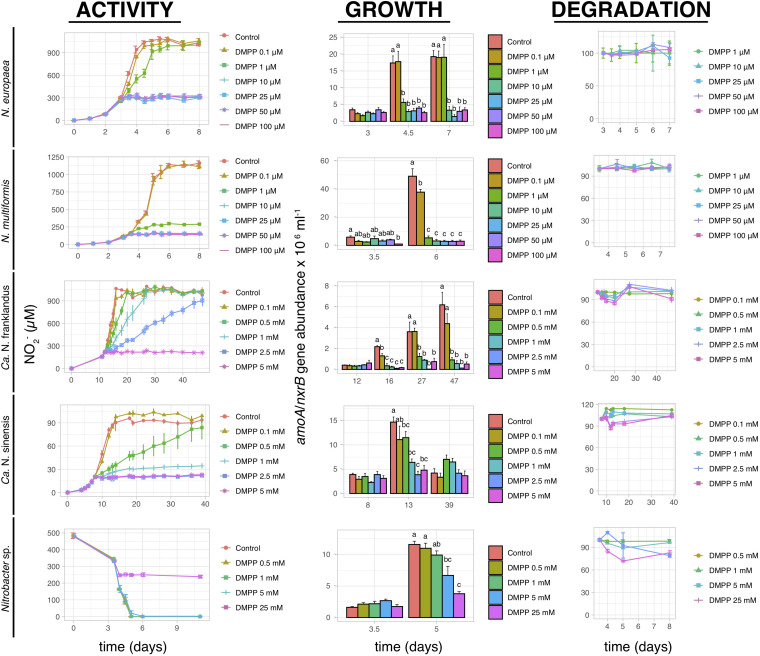
The effect of different concentrations of DMPP on the activity and growth of AOB *N. europaea* and *N. multiformis*, AOA “*Ca.* N. franklandus” and “*Ca.* N. sinensis” and NOB *Nitrobacter* sp. NHB1, determined by nitrite production, or consumption and the abundance of *amoA* or *nxrB* genes. The degradation pattern of DMPP applied over a range of concentrations is also presented. Error bars represent standard error of the mean of biological triplicates. Within each time point bars designated by different lower-case letters are significantly different at the 5% level.

3,4-dimethylpyrazole phosphate induced complete inhibition of nitrite production by *N. europaea* and *N. multiformis* at concentrations ≥10 μM and ≥1 μM, respectively, (Figure 5). The pattern of AOB growth inhibition was congruent with NO_2_^–^ production, except for a weak (22.9 ± 3.9%) but significant (*p* < 0.05) inhibition of *N. multiformis* growth at 0.1 μM compared to the control. Conversely, DMPP significantly inhibited the activity of both AOA isolates at higher concentrations of ≥0.5 mM (*p* ≤ 0.05), with complete inhibition of “*Ca.* N. franklandus” and *Ca.* N. sinensis” occurring only at 5 mM and ≥1 mM, respectively, (Figure 5). In certain cases, the impact of DMPP on nitrite production was not concomitant with growth patterns, with DMPP concentrations ≥0.5 mM inducing a persistent reduction in *amoA* gene abundance of “*Ca.* N. franklandus” (Figure 5). DMPP completely inhibited nitrite oxidation by *Nitrobacter* sp. NHB1 only at the highest tested concentrations of 25 mM, while its growth was significantly suppressed at concentrations ≥5 mM (*p* < 0.05; Figure 5). DMPP was rather persistent in liquid cultures with its DT_50_ values ranging from 14.34 to >1000 days without a dose-dependent degradation pattern (Figure 5 and Supplementary Table 1).

### Comparison of NIs Activity Based on Calculated EC_50_ Values

**FIGURE 6:**
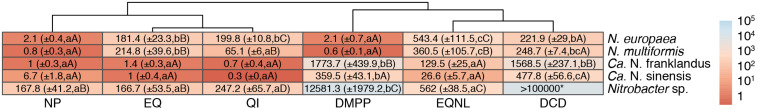
Mean EC_50_ values (μM) of the tested nitrification inhibitors (NIs) calculated based on their inhibitory activity on the ammonia or nitrite oxidation capacity of AOA, AOB, and NOB isolates. Standard errors of the mean values (denoted by±) are given in brackets. Upper case letters indicate significant differences (*p* < 0.05) between microorganisms for each individual NI, and lower-case letters indicate significant differences (*p* < 0.05) between NIs for each tested microorganism. The asterisk denotes that no EC_50_ could be descent from the statistical analysis. Dendrograms based on the Euclidean distances and the complete linkage clustering method using log transformed mean EC_50_ values are presented for identifying NIs. The table is color-coded by orders of magnitude for EC_50_ values according to the color legend.

The two AOB isolates showed equivalent EC_50_ values for the NIs tested (Figure 6) with the exception of EQ derivatives, where significantly higher EC_50_ values were observed for *N. europaea* compared to *N. multiformis* for both QI and EQNL (*p* < 0.001). DMPP and NP were the most potent inhibitors of *N. europaea*, followed by EQ, QI, and DCD which were not significantly different, with EQNL being the weakest inhibitor (EC_50_ = 181.4 ± 23.3 μM). For *N. multiformis*, DMPP, NP, and QI were equally effective inhibitors, followed by EQ, DCD, and EQNL. The two AOA strains exhibited contrasting responses, with “*Ca.* N. franklandus” having decreased sensitivity to DCD and DMPP compared to *“Ca.* N. sinensis.” EQ, its derivatives and NP were equally effective inhibitors of both AOA isolates, with QI having the lowest EC_50_ values (0.3 ± 0.0–0.7 ± 0.4 μM), while DCD and DMPP were the weakest AOA inhibitors (Figure 6). EQ, its derivatives and NP were equally suppressive toward *Nitrobacter* sp. NHB1, while DMPP and DCD showed no appreciable inhibition.

## Discussion

This study is the first to investigate the inhibitory effect of EQ, a novel NI of potential agricultural relevance, and its oxidation derivatives, QI and EQNL, on soil nitrifiers grown in pure cultures and demonstrate greater inhibition of all three compounds on AOA compared to AOB isolates. In all cultures, EQ was rapidly transformed to QI and EQNL, with the former being the major but least persistent derivative, while the latter being the minor but more persistent derivative, and was consistent with previous studies in soil (Karas et al., 2015; Papadopoulou et al., 2016). Considering that (i) in all cultures, QI showed equivalent or higher inhibitory activity compared to its parent compound, and (ii) in EQ-amended cultures, QI was formed at concentrations equal or higher than those expected to induce an inhibitory effect on the AO tested, and EQNL was formed at levels substantially lower than those expected to result in an inhibitory effect on the AO tested (Figure 1 and Supplementary Table 2), we suggest that QI is the main determinant for the persistent inhibitory effect of EQ on AO and NOB, in line with our previous soil studies (Papadopoulou et al., 2016). The higher inhibition potential of QI for AOA compared to AOB isolates, contradicts our previous soil studies, where equivalent inhibitory effects against both groups were observed. Although direct comparisons between soil and culture studies cannot be made, we suggest that the concentrations of QI formed in soil samples (up to 86.1 μmol Kg^–1^ dwt soil) probably reached or exceeded its inhibition threshold levels for both AO groups.

In contrast to EQ and its derivatives, DCD and DMPP exhibited higher inhibitory activity toward AOB isolates as observed by Shen et al. (2013) who reported greater inhibition by DCD on *N. multiformis* compared to the AOA *N. viennensis*. Of these two NIs, DMPP showed greater inhibitory activity toward both AOB isolates. Data on the inhibitory activity of DMPP toward soil-derived cultures of AOB and AOA strains are scarce. Liu et al. (2019) recently reported greater inhibition by DMPP (EC_50_ = 448 μM) compared to DCD (EC_50_ = 947.1 μM) to “*Ca.* Nitrosocosmicus agrestis,” a soil strain closely related to “*Ca*. N. franklandus.” Unlike DMPP, there are several reports on the inhibitory activity of DCD on soil AOA and AOB cultures with DCD strongly inhibiting *Nitrososphaera* sp. JG1 (Kim et al., 2012) and *Ca.* Nitrosarchaeum koreensis MY1 (Jung et al., 2011) at 0.5 mM which was in the same range to the two AOA strains examined here. Lehtovirta-Morley et al. (2013) showed that DCD induced a significant inhibition of “*Ca.* Nitrosotalea devanaterra” at 1 mM, compared to 0.5 mM needed for the inhibition of “*Ca*. N. sinensis” in our study. Others reported EC_50_ values of 950 μM for “*Ca*. N. agrestis” (Liu et al., 2019), and 940.6 μM for *N. viennensis* (Shen et al., 2013) compared to 1568.5 μM observed here for “*Ca.* N. franklandus.” For AOB, Shen et al. (2013) reported an EC_50_ of 80.3 μM for DCD on *N. multiformis* compared to 248.7 μM observed in our study for the same strain. Although there are no obvious differences between the two studies explaining this variation, the salt and concentration of ammonium was different which may have affected the growth characteristics of *N. multiformis*.

Nitrapyrin was the only tested NI that showed an equivalent and strong inhibitory effect toward both AOB and AOA isolates, suppressing their activity at concentrations ≥0.5–5 μM and ≥1–5 μM, respectively. This in line with previous studies which showed inhibition of AOB (*Nitrosomonas* sp., *Nitrosospira* sp., *Nitrosolobus* sp., *N. europaea, N. multiformis)* and AOA strains (*Nitrososphaera* sp. JG1, *Nitrosarchaeum koreensis* MY1) at levels varying from 0.86 μM for AOB (Bélser and Schmidt, 1981) to 10 μM for both AOB and AOA (Jung et al., 2011; Kim et al., 2012; Martens-Habbena et al., 2015). Comparison with other AOA isolates indicates that inhibition characteristics are similar between strains belonging to the same phylogenetic group. For example, Lehtovirta-Morley et al. (2013) demonstrated that NP halted the activity of “*Ca.* N. devanaterra ND1” at concentrations ≥10 μM compared to ≥5 μM for “*Ca*. N. sinensis (ND2)” in our study, while Liu et al. (2019) reported an EC_50_ of 0.6 μM for “*Ca*. N. agrestis” compared to 1 μM for “*Ca.* N. franklandus” in our study. However, in contrast to our findings for NP inhibition of *N. multiformis* (EC_50_ 0.8 ± 0.3 μM), Shen et al. (2013) reported a much weaker inhibitory effect for the same strain (EC_50_ > 173 μM). In addition to the minor differences in cultivation conditions between the two studies, Shen et al. added solid NP directly into the cultures to achieve concentrations in the range of 40–173 μM, with the highest level corresponding to the upper limit of NP water solubility at 20°C (40 mg L^–1^), entailing a risk for precipitation of the active compound.

The considerable range in the inhibitory concentrations of the tested NIs may indicate differences in their mode of action not considered previously. For example, DCD, DMPP, and NP, all considered as Cu-chelators, varied in their ability to inhibit AOA (Lehtovirta-Morley et al., 2013; Shen et al., 2013; Liu et al., 2019). In addition, NP has also been proposed to function as an alternative AMO substrate, generating 6-chloropicolinic acid which irreversibly deactivates ammonia oxidation (Vannelli and Hooper, 1992). This inhibitory mechanism proposed for NP may offer an explanation for its rather universal inhibitory activity toward AOA and AOB. Both EQ and its derivatives possess high-antioxidative capacity acting as free radical scavengers (Błaszczyk et al., 2013). As EQ and its degradation product QI showed similar inhibitory effects to NO-scavengers (e.g., PTIO; Martens-Habbena et al., 2015), their efficiency against AOA may be due to a similar mode of action. Alternatively, as QI is a strong antioxidant, it could be involved in oxidative stress-related cell disruption particularly in AOA, with AOB being capable of coping with oxidative stress using catalases, enzymes which are largely absent in AOA (Kim et al., 2016).

In addition to the contrasting differences in sensitivity between AOA and AOB to all NIs tested (except for NP), we also observed differences in the sensitivity between the two AOA or two AOB strains examined. For QI and EQNL, *N. multiformis* was consistently more sensitive than *N. europaea*, and for DCD and DMPP, “*Ca.* N. sinensis” was consistently more sensitive than “*Ca.* N. franklandus.” Studies on the comparative sensitivity of AOB isolates to chemicals, including NIs, are scant. Brandt et al. (2001) reported a higher sensitivity of *N. multiformis* over *N. europaea* to linear alkylbenzene sulfonate surfactants. The different sensitivity of the two AOB isolates to EQ derivatives is probably related to differences in the physiology of these isolates. Comparative genomic and proteomic analysis of *N. europaea* and *N. multiformis* showed that the two strains possess a largely different set of stress response proteins, alkyl hyperoxide reductase *vs* superoxide dismutase and rubrerythrin, respectively, that might exhibit different efficiencies to stress imposed by QI and EQNL (Zorz et al., 2018). Alternatively, *N. europaea* has a greater array of membrane protein transporters, potentially enabling a greater efflux of toxic chemicals (Zorz et al., 2018).

The different sensitivities of the two tested AOA isolates to DCD and DMPP are also associated with the contrasting ecophysiologies (Lehtovirta-Morley et al., 2014, 2016). In line with our findings, previous studies comparing the two strains reported a higher sensitivity of “*Ca*. N. sinensis” to both simvastatin (Zhao et al., 2020) and 3,5-dichloraniline (Vasileiadis et al., 2018). The higher tolerance of “*Ca*. N. franklandus” to DCD and DMPP might be associated with its capacity to produce extracellular polymeric substances (EPS) leading to aggregate formation that block the hydrophilic NIs DCD and DMPP of accessing the surface of cells engulfed into hydrophobic EPS (Gao et al., 2007). This production of EPS is a feature shared by all *Ca*. Nitrosocosmicus isolates (Jung et al., 2016; Lehtovirta-Morley et al., 2016; Sauder et al., 2017; Alves et al., 2019; Liu et al., 2019) and has been reported as a protection mechanism of AOB against NIs (Powell and Prosser, 1991).

The comparative analysis of the inhibitory range of the tested NIs highlights the practical implications of our findings. The two most widely used NIs, DCD, and DMPP, showed high inhibitory activity only to AOB, the latter being the most potent AOB inhibitor together with NP. While NP is the only NI currently used in agriculture that demonstrates equal inhibition of both AOB and AOA, it is not currently registered for use in Europe. These findings have serious practical implications for nitrification inhibition in agricultural soils with acidic to neutral pH, which constitute 30% of the World’s soils (pH < 5.5) and a large fraction of European agricultural soils (mean soil pH = 5.8; Fabian et al., 2014), and where ammonia oxidation is often dominated by AOA (Zhao et al., 2020). Differences in the inhibition thresholds between AOA and AOB could affect agricultural practice, as AOA may contribute to nitrogen fertilizer loss under conditions when AOB are inhibited (Hink et al., 2018). Conversely, universal inhibitory effects on both AOB and AOA, and perhaps comammox bacteria recently reported to be inhibited by NP (primarily), DCD and DMPP in soil microcosm studies (Li et al., 2019b), suggest that nitrification inhibition would not be compromised by functional redundancy. Alternatively, the use of mixtures of NIs exhibiting complementary activity against different AO groups or targeting different parts of the ammonia oxidation pathway could be as efficient as using broad range NIs. In this regard, the potential agricultural use of EQ as a novel NI, applied alone or in combination with NIs selective to AOB (i.e., DMPP) could be promising, considering its low cost (equivalent or lower than that of DCD and NP; Błaszczyk et al., 2013), and its unique feature to be transformed in soil to QI, a highly potent inhibitor of AOA and whose activity to AOB is comparable with that of NIs currently used in agricultural settings such as DCD. Although extrapolating from pure culture tests to predicted effects in soil should be performed with caution, based on our liquid culture assays, a soil concentration of 10 mg Kg^–1^ of EQ (corresponding to 14.4 Kg ha^–1^, assuming incorporation of the NI to the top 5 cm of the soil profile of a field site of 1 ha, and soil bulk density 1.3 g cm^3^) could have a universal inhibitory effect on both AOA and AOB, while 0.06 mg Kg^–1^ (<0.1 Kg ha^–1^) would be required to achieve effective inhibition AOA only. This is consistent with earlier studies that showed that EQ, when applied in soil at concentration levels (50 mg Kg^–1^) simulating a wastewater disposal scenario, resulted in inhibition of both AOA and AOB (Papadopoulou et al., 2016). Such application rates are in the same range as those of established NIs, providing the first evidence for the feasibility of EQ use in an agricultural setting. On-going studies will determine the effective dose rates of EQ under full-scale agricultural conditions.

There is less known about the direct effects of NIs on NOB, despite their important regulatory function in the overall nitrification process (Daims et al., 2016). NOB are closely associated with AOs and their activity results in the rapid conversion of potentially toxic nitrite to nitrate (Matsumoto et al., 2009), an important nitrogen source for plants and aerobic soil microorganisms (Koch et al., 2015). In disturbed agricultural ecosystems such as fertilized soils, NOB-derived nitrate production contributes to N losses and environmental pollution through nitrate leaching and subsequent denitrification processes (Raun and Johnson, 1999). We demonstrated that DMPP and DCD were not active against *Nitrobacter* sp. NHB1, in contrast to NP, EQ, and its derivatives which were suppressive to the tested isolate in μM concentration levels. Our study was the first to provide data regarding the impact of DCD, DMPP, EQ, and its derivatives on a pure NOB culture, while NP previously applied at rates up to 50 μM did not inhibit the nitrite-oxidizing activity of the widely distributed *Nitrobacter agilis* (Matsuba et al., 2003), in line with our results.

The impact of NP, EQ, and its derivatives on the activity of both AOs and NOB could affect the total nitrogen balance, and the direction and degree of nitrogen transformation during the nitrification process. This could have serious practical and ecological implications in cases where NIs inhibit NOB to a greater extent than AOs. This would lead to possible NO_2_^–^ accumulation in soil and increased NO_2_^–^ driven N_2_O production (Venterea et al., 2015) with reciprocal effects for the environment and plant productivity. However, our findings suggest a lower (DCD, NP, DMPP, and QI) or equivalent (EQ) inhibition potential of NIs against *Nitrobacter* sp. compared to AOs (Figure 6). Further studies extended to other NOB, including the widely distributed and diverse *Nitrospira*-like bacteria, would determine the full inhibitory potential of NIs on soil NOB.

In parallel, we determined the degradation and transformation of the tested NIs to identify potential links between the duration of exposure (persistence) and the effects observed. The total residues of EQ showed limited persistence in the AOA and NOB cultures (DT_50_ = 2.4–8.7 days), and low to moderate persistence in the AOB cultures (DT_50_ = 8.7–60.1 days), a difference most likely attributed to abiotic factors such as medium pH (acidic for *Ca.* N. sinensis and *Nitrobacter sp.* vs. alkaline for *N. europaea* and *N. multiformis*) rather than an enzymatic transformation, considering the autotrophic lifestyle of the tested isolates (Kim et al., 2016), the lack of genetic repertoire for the catabolism of organic pollutants (Chain et al., 2003; Norton et al., 2008), and the recalcitrance of EQ to biotic degradation under aerobic and anaerobic conditions (Shah et al., 2005). However, a direct interaction of these compounds with the tested organisms cannot be fully excluded. The three commercial NIs showed remarkably different stability in the liquid cultures. DCD showed moderate to high persistence (DT_50s_ = 44.5 to >1000 days) with the lowest DT_50_ values observed in the liquid cultures of *Nitrobacter* sp. NHB1, suggesting a potential interaction with this strain. Microbial mineralization of DCD by pure cultures of soil isolated bacteria has been previously reported (Hallinger et al., 1990; Hawser and Haselwandter, 1990). Later studies suggested an enzymatic hydrolysis of the NI catalyzed by microbial ureases (Estermaier et al., 1992). This was not confirmed in our study since no significant differences in the stability of DCD between urease-positive (*N. multiformis*, *Ca.* N. franklandus) and urease-negative (*N. europaea*, *Ca.* N. sinensis, and *Nitrobacter* sp. NHB1) AOs were observed. Abiotic parameters such as temperature or pH could potentially influence the stability of DCD in soil (Amberger, 1986; Hallinger et al., 1990; Kelliher et al., 2008). However, in our studies we did not observe any clear effects of temperature (28°C for AOB and NOB, and 35°C for AOA) and pH (7.5–8.0 for AOB and *Ca.* N. franklandus, and 5.2 for *Ca.* N. sinensis and *Nitrobacter* sp. NHB1) on DCD stability in our liquid culture conditions. NP degraded rapidly (DT_50_ = 0.12–12.5 days) in all liquid cultures. In contrast, DMPP showed a high persistence in all liquid cultures, except of *Nitrobacter* where a great variation in its persistence was evident. Considering that *Nitrobacter* sp. NHB1 and AOA were cultured in media of similar content and pH, the above variation was most probably driven by interaction between DMPP and the bacterium. Genomic analysis suggested limited catabolic capacity of aromatic compounds by *Nitrobacter* strains (Starkenburg et al., 2008), although some studies have shown an appreciable degradation of crude oil by *Nitrobacter* (Jong and Okpokwasili, 2012). Overall, we did not observe any clear correlations between NIs persistence and inhibition potency, except for the lower persistence of DCD and DMPP in the liquid cultures of *Nitrobacter* sp. NHB1 which coincided with the limited activity of these NIs to the bacterium.

## Conclusion

We compared the inhibition potential of EQ, a novel NI, and those currently used in agricultural practice, on the activity and growth of soil-derived AOA, AOB, and NOB isolates grown in liquid culture. EQ, and primarily its major derivative QI, showed high potency against AOA, in contrast to DCD and DMPP (the only NIs currently registered for use in Europe) which were inhibitory to AOB only. Conversely, NP showed an equally high inhibitory activity against both AOA and AOB isolates. EQ, QI, and NP were the most potent AOA and NOB inhibitors, unlike DCD and DMPP, which demonstrated no activity. DMPP and NP were the most potent AOB inhibitors, with EQ, QI, and DCD showing lower but still appreciable inhibitory activity. Our study (i) offers benchmarking knowledge of the activity range of currently used in agriculture and potentially new NIs to soil AO and *Nitrobacter* NOB, whose response to NIs were unknown, (ii) introduces a novel potential NI, EQ, which possesses desirable characteristics, including transformation into a highly potent NI (QI) characterized by high inhibitory activity against AOA compared to currently registered NIs in Europe, and (iii) demonstrates the different sensitivity of AOA and AOB to NIs, which indicates that novel strategies for effective nitrification inhibition should rely on new broad-range NIs, or more likely, mixtures of NIs with complementary activity against different nitrifier groups. Future work will focus on the elucidation of EQ and QI inhibitory mechanisms, and on the evaluation of their environmental and agronomic performance under diverse edaphic and climatic conditions and on soils with different microbial communities.

## Data Availability Statement

The raw data supporting the conclusions of this article will be made available by the authors, without undue reservation.

## Author Contributions

EP planned, performed, and supervised the experiments, carried out data analysis, drafted and revised the final manuscript. EB, EL, EA, and AK participated in parts of the culture inhibition assays. SV performed the modeling for the calculation of EC_50_ and DT_50_ values, constructed and edited data figures, and reviewed the manuscript. CT planned and participated in part of the culture inhibition assays. UM-S supervised the chemical synthesis of EQ derivatives and reviewed the manuscript. GN provided nitrifying cultures, supervised part of the culture inhibition assays, and reviewed the manuscript. DK conceived the experimental rationale, co-supervised the experiments, and reviewed the manuscript. All authors contributed to the article and approved the submitted version.

## Conflict of Interest

The authors declare that the research was conducted in the absence of any commercial or financial relationships that could be construed as a potential conflict of interest.

## Supplementary Material

Supplementary Figure 1 The chemical structures of the tested nitrification inhibitors (NIs).

Supplementary Figure 2 The effect of different concentrations of EQNL on the activity and growth of AOB *N. europaea* and *N. multiformis*, AOA “*Ca.* N. franklandus” and “*Ca.* N. sinensis” and NOB *Nitrobacter* sp. NHB1, determined by nitrite production or consumption and the abundance of *amoA* or *nxrB* genes. The degradation pattern of EQNL applied at a range of concentrations in the liquid cultures of the nitrifying isolates, is also presented. Error bars represent the standard error of the mean of triplicate cultures. Within each time point bars designated by different lower-case letters are significantly different at the 5% level.

Supplementary Figure 3 The degradation and transformation patterns of EQ in the liquid cultures of “*Ca.* N. franklandus” (a, b) and “*Ca.* N. sinensis” (c, d) amended with 4.6 μM (a, c) and 46 μM of EQ (b, d). Each value is the mean of triplicates ± standard error. Bars designated by one asterisk show the concentration of EQ and its oxidative derivatives at the onset of inhibition, while bars designated by two asterisks indicate the time point when maximum QI concentrations were observed.

Supplementary Table 1 DT_50_ values (days) of the different nitrification inhibitors (NIs) tested per nitrifying isolate and NI concentrations used. DT_50_ values were calculated by fitting the best fitting kinetic model to the degradation data.

Supplementary Table 2 Mean concentrations ± standard errors (μM) of QI and EQNL formed in liquid cultures of nitrifying isolates amended with EQ at the (i) onset of inhibition, and (ii) time of detection of their maximum concentration levels. The timepoint (days) at which each measurement was taken is given in brackets.

## Abbreviations

NIs: nitrification inhibitors
EQ: ethoxyquin
QI: 2,6-dihydro-2,2,4-trimethyl-6-quinone imine
EQNL: 2,4-dimethyl-6-ethoxyquinoline
DCD: dicyandiamide
NP: nitrapyrin
DMPP: 3,4-dimethylpyrazole phosphate
AOB: ammonia-oxidizing bacteria
AOA: ammonia-oxidizing archaea
AO: ammonia-oxidizers
NOB: nitrite-oxidizing bacteria
comammox: complete ammonia-oxidizing bacteria
AMO: ammonia monooxygenase
EPS: extracellular polymeric substances.
