# Supplementary figures and images for: Comparison of Novel and Established Nitrification Inhibitors Relevant to Agriculture on Soil Ammonia- and Nitrite-Oxidizing Isolates
Source: Front Microbiol. 2020 Nov 4;11:581283. doi: 10.3389/fmicb.2020.581283 (PMC7672009; doi:10.3389/fmicb.2020.581283)

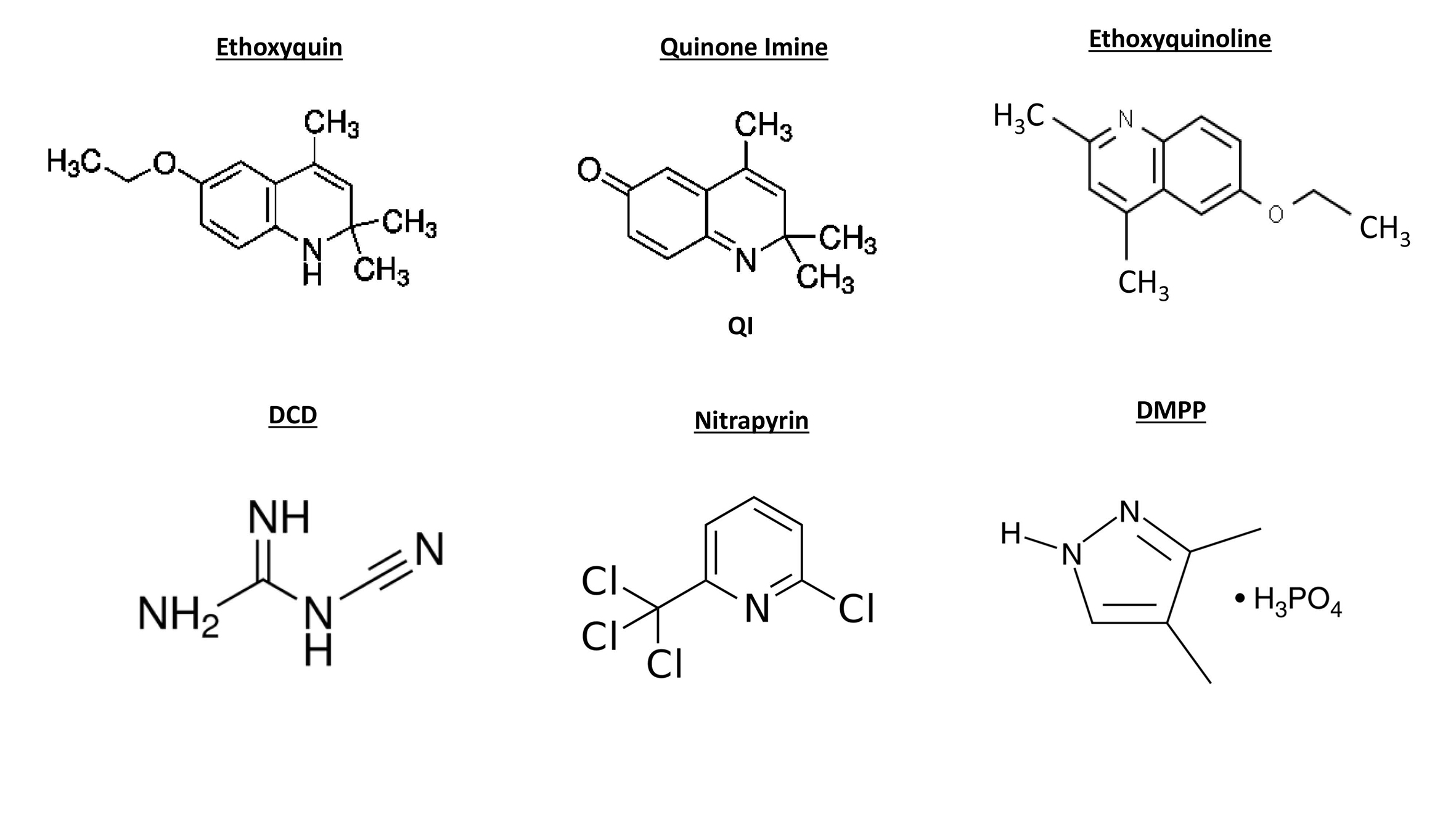

Supplement: Supplementary Figure 1 — The chemical structures of the tested nitrification inhibitors (NIs). [file Image_1.JPEG]

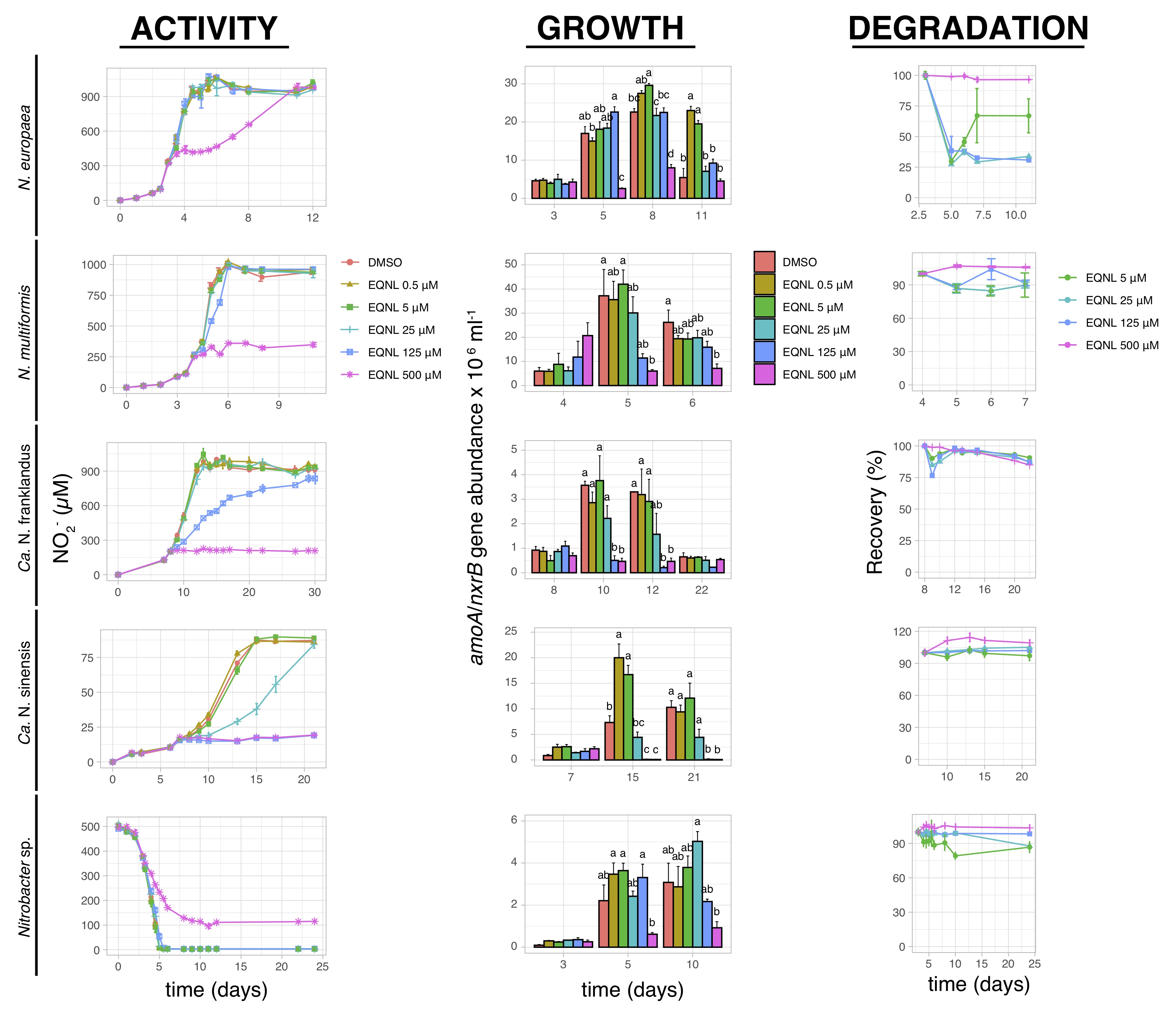

Supplement: Supplementary Figure 2 — The effect of different concentrations of EQNL on the activity and growth of AOB N. europaea and N. multiformis, AOA “Ca. N. franklandus” and “Ca. N. sinensis” and NOB Nitrobacter sp. NHB1, determined by nitrite production or consumption and the abundance of amoA or nxrB genes. The degradation pattern of EQNL applied at a range of concentrations in the liquid cultures of the nitrifying isolates, is also presented. Error bars represent the standard error of the mean of triplicate cultures. Within each time point bars designated by different lower-case letters are significantly different at the 5% level. [file Image_2.JPEG]

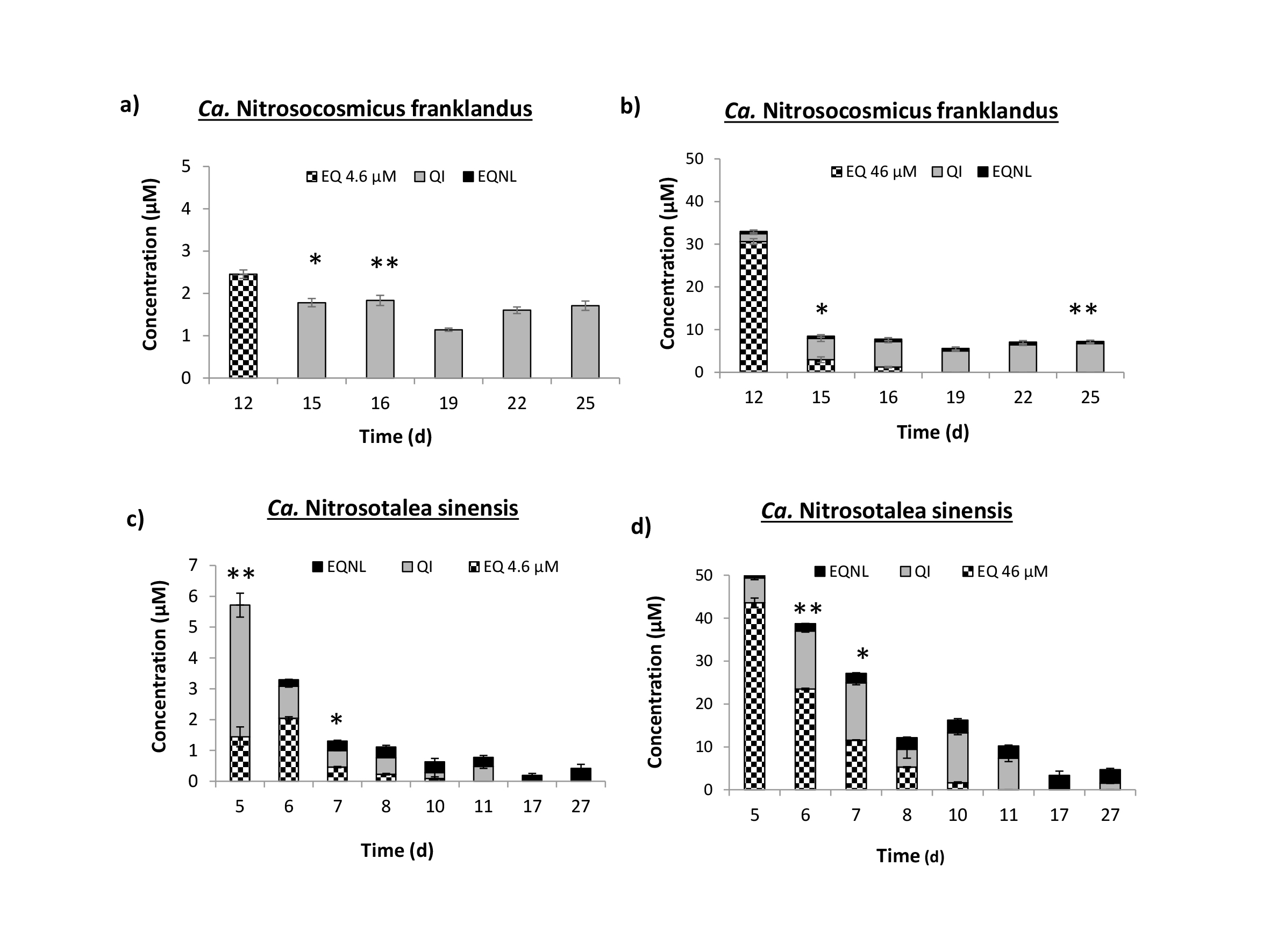

Supplement: Supplementary Figure 3 — The degradation and transformation patterns of EQ in the liquid cultures of “Ca. N. franklandus” (a, b) and “Ca. N. sinensis” (c, d) amended with 4.6 μM (a, c) and 46 μM of EQ (b, d). Each value is the mean of triplicates ± standard error. Bars designated by one asterisk show the concentration of EQ and its oxidative derivatives at the onset of inhibition, while bars designated by two asterisks indicate the time point when maximum QI concentrations were observed. [file Image_3.JPEG]
