# Supplementary material for: Comparison of Novel and Established Nitrification Inhibitors Relevant to Agriculture on Soil Ammonia- and Nitrite-Oxidizing Isolates
Source: Front Microbiol. 2020 Nov 4;11:581283. doi: 10.3389/fmicb.2020.581283 (PMC7672009; doi:10.3389/fmicb.2020.581283)
Supplement: Supplementary Table 1 — DT50 values (days) of the different nitrification inhibitors (NIs) tested per nitrifying isolate and NI concentrations used. DT50 values were calculated by fitting the best fitting kinetic model to the degradation data. [file Table_1.docx]

**Supplementary Table S1.** DT_50_ values (days) of the different nitrification inhibitors (NIs) tested per nitrifying microorganism and NI concentrations used. DT_50_ values were calculated by fitting the degradation data to the best fitting kinetic model. The first order kinetic model provided the best fit to the experimental data in most cases. In all other cases the model providing the best fit is indicated (i.e. HS: Hockey-Stick model; FOMC: First order multicompartment model; DFOP: Double first order in parallel model)

| **Nitrification Inhibitor** | **Concentration (μΜ)** | ***Nitrosomonas europaea*** | ***Nitrosospira multiformis*** | ***"Ca.* N. franklandus"** | ***"Ca.* N. sinensis"** | ***Nitrobacter sp.* NHB1** |
| --- | --- | --- | --- | --- | --- | --- |
| **Total**  **Ethoxyquine (EQ)**  **residues** | **46** | 8.68 | 11.6 | 4.96 | 2.25 | 3.66 |
|  | **460** | 48.5 | 60.1 | 8.72 | 2.42 | 2.06 |
| **Quinone Imine (QI)** | **2.7** | 0.05 | 0.52 | 1.39 | 1.52 | 0,90 |
|  | **27** | 0.42 | 0.92 | 1.26 | 0.85 | 0,54 |
|  | **135** | 1.19 | 1.32 | 1.74 | 1.38 | 0.92 |
|  | **270** | 1.91 | 2.47 | 2.68 | 1.92 | 1.18 |
|  | **540** | 3.55 | 5.65 | 4.50 | 2.93 | 2.23 |
| **Ethoxyquinoline (EQNL)** | **5** | 7.31 | >1000 | 290.8 | 699.9 | 123.5 |
|  | **25** | 10.92 | 18.3 | 289.8 | >1000 | 119.6 |
|  | **125** | 12.16 | 73.9 | 400 | >1000 | >1000 |
|  | **500** | 82.5 | >1000 | 86.9 | >1000 | >1000 |
| **Dicyandiamide**  **(DCD)** | **25** | 66.9 | 111.9 |  |  |  |
|  | **50** | 402.3 | >1000 |  |  |  |
|  | **100** | >1000 | 109.6 |  |  |  |
|  | **250** | 106.4 | 78.5 | >1000 | 225.9 |  |
|  | **500** | 78.3 | 55.5 | 494.1 | 356 |  |
|  | **1000** |  |  | >1000 | >1000 |  |
|  | **2000** |  |  |  |  | 46.9 |
|  | **2500** |  |  | 997.5 | >1000 |  |
|  | **4000** |  |  |  |  | 47.8 |
|  | **5000** |  |  | >1000 | >1000 |  |
|  | **20000** |  |  |  |  | 45.9 |
|  | **100000** |  |  |  |  | 84.0 |
| **Nitrapyrin**  **(NP)** | **1** |  |  | 1.43 | 1.28 |  |
|  | **5** | 1.53 | 0.82^HS^ | 0.12^FOMC^ | 1.90 |  |
|  | **10** |  |  |  |  | 2.43 |
|  | **20** |  |  |  |  | 3.24 |
|  | **25** | 2.85 | 0.76^HS^ | 0.13 ^DFOP^ | 1.85 |  |
|  | **50** | 3.40 | 0.56^FOMC^ |  |  |  |
|  | **100** | 3.24 | 4.01 | 0.15 ^DFOP^ | 2.10 | 4.02 |
|  | **500** |  |  |  |  | 12.5 |
| **DMPP** | **1** | >1000 | >1000 |  |  |  |
|  | **10** | >1000 | >1000 |  |  |  |
|  | **25** | >1000 | >1000 |  |  |  |
|  | **50** | >1000 | >1000 |  |  |  |
|  | **100** | >1000 | >1000 | >1000 | >1000 |  |
|  | **500** |  |  | >1000 | >1000 | 46.9 |
|  | **1000** |  |  | >1000 | >1000 | 37.8 |
|  | **2500** |  |  | >1000 | >1000 |  |
|  | **5000** |  |  | >1000 | >1000 | >1000 |
|  | **25000** |  |  |  |  | 14.34 |
