# Supplementary material for: Comparison of Novel and Established Nitrification Inhibitors Relevant to Agriculture on Soil Ammonia- and Nitrite-Oxidizing Isolates
Source: Front Microbiol. 2020 Nov 4;11:581283. doi: 10.3389/fmicb.2020.581283 (PMC7672009; doi:10.3389/fmicb.2020.581283)
Supplement: Supplementary Table 2 — Mean concentrations ± standard errors (μM) of QI and EQNL formed in liquid cultures of nitrifying isolates amended with EQ at the (i) onset of inhibition, and (ii) time of detection of their maximum concentration levels. The timepoint (days) at which each measurement was taken is given in brackets. [file Table_2.DOCX]

**Supplementary Table S2.** Mean concentrations ± standard errors (μΜ) of QI and EQNL formed in the liquid cultures of nitrifying isolates amended with EQ (i) at the onset of inhibition, (ii) at time of detection of maximum concentration levels. The timepoint (days) at which each measurement was taken is given in brackets.

| **Ethoxyquin treatment** | | **Concentration (μΜ)** | | | | |
| --- | --- | --- | --- | --- | --- | --- |
|  |  | **Inhibition Onset** | | | **Maximum concentration formed** | |
|  |  | **QI** | **EQNL** | **QI** | | **EQNL** |
| **AOB** | *N. europaea*- 460 μΜ | 95.4 ±2.1 (7d) | 3.42±0.2 (7d) | 170.2±1.4 (14d) | | 3.42±0.2 (7d) |
|  | *N. multiformis*- 460 μΜ | 36.6±3.4 (8d) | 8.98±0.9 (8d) | 167.6±16.1 (16d) | | 10.3±0.8 (14d) |
| **AOA** | “*Ca.* N. franklandus” – 460 μΜ | 74.9±0.9 (15d) | 8.80±0.1 (15d) | 96.3±1.5 (19d) | | 10.3±0.2 (22d) |
|  | “*Ca.* N. franklandus” – 46 μΜ | 4.97±0.7 (15d) | 0.61±0.3 (15d) | 6.75±0.1 (25d) | | 0.61±0.4 (22d) |
|  | “*Ca.* N. franklandus” – 4.6 μΜ | 1.78±0.1 (15d) | 0.0 (0.0) | 1.83±0.1 (16d) | | 0.0 (0.0) |
|  | “*Ca.* N. sinensis” – 460 μΜ | 132.6±1.5 (7d) | 16.9±0.4 (7d) | 189.8±13.8 (10d) | | 27.4±1.1 (17d) |
|  | “*Ca.* N. sinensis” – 46 μΜ | 13.3±0.4 (7d) | 2.3±0.1 (7d) | 13.5±0.7 (6d) | | 3.4±1.0 (17d) |
|  | “*Ca.* N. sinensis” – 4.6 μΜ | 1.0±0.0 (7d) | 0.20±0.0 (7d) | 4.3±0.4 (5d) | | 0.4±0.1 (27d) |
| **NOB** | *Nitrobacter* sp. - 460 μΜ | 50.8±13.1 (4d) | 11.5±0.9 (4d) | 114.5±1.8 (9d) | | 14.2± 0.6 (3d) |
